# Supplementary material for: Major acute cardiovascular events after dengue infection–A population-based observational study
Source: PLoS Negl Trop Dis. 2022 Feb 7;16(2):e0010134. doi: 10.1371/journal.pntd.0010134 (PMC8853534; doi:10.1371/journal.pntd.0010134)
Supplement: S1 Table — (DOCX) [file pntd.0010134.s001.docx]

**S1 Table. Baseline characteristics of patients with onychomycosis and influenza infection who had a MACE within the observation period**

| Characteristics | **Influenza** | | **Onychomycosis** | |
| --- | --- | --- | --- | --- |
|  | N | % | N | % |
| Age |  |  |  |  |
| 0-39 years | 196 | 4.29 | 245 | 1.66 |
| 40-59 years | 627 | 13.72 | 2,733 | 18.48 |
| ≥60 years | 3,743 | 81.90 | 11,812 | 79.86 |
| Missing | 4 | 0.09 | 0 | 0.00 |
| Sex |  |  |  |  |
| Male | 2,609 | 57.09 | 9,115 | 61.63 |
| Female | 1,948 | 42.63 | 5,660 | 38.27 |
| Hypertension | 13 | 0.28 | 15 | 0.10 |
| Diabetes mellitus | 3,121 | 68.29 | 10,444 | 70.62 |
| Dyslipidemia | 1,857 | 40.63 | 5,652 | 38.22 |
| MACE before observation period | 1,050 | 22.98 | 4,347 | 29.39 |
| Hemorrhage stroke | 2,824 | 61.79 | 8,240 | 55.71 |
| Ischemic stroke | 165 | 3.61 | 623 | 4.21 |
| Acute myocardial infarction | 899 | 19.67 | 3,279 | 22.17 |
| Heart failure | 409 | 8.95 | 1,506 | 10.18 |
| MACE after observation period | 1,351 | 29.56 | 2,832 | 19.15 |
| Hemorrhage stroke | 3,328 | 72.82 | 10,097 | 68.27 |
| Ischemic stroke | 182 | 3.98 | 745 | 5.04 |
| Acute myocardial infarction | 769 | 16.83 | 3,464 | 23.42 |
| Heart failure | 519 | 11.36 | 2,061 | 13.94 |

MACE, major adverse cardiovascular events
