# Supplementary material for: Major acute cardiovascular events after dengue infection–A population-based observational study
Source: PLoS Negl Trop Dis. 2022 Feb 7;16(2):e0010134. doi: 10.1371/journal.pntd.0010134 (PMC8853534; doi:10.1371/journal.pntd.0010134)
Supplement: Attachment — Submitted filename: Reply to All 20211111.pdf [file pntd.0010134.s002.pdf]

Dear Editor-in-Chief,

Thank you for your interest in our manuscript entitled, "*Major acute cardiovascular events after dengue infection– A population-based observational study*".

We would like to thank you and reviewers for the comments and constructive suggestions. Our apologies for the typographical errors. They have all been corrected according to the reviewer's suggestions. Changes have been made to the manuscript and are highlighted with tracking system accordingly.

We hope our answers have fully addressed the questions of the reviewer and our revised manuscript will find your approval. For your comments, we would like to respond point by point as follows.

Thank you very much for your consideration. I look forward to hearing from you.

Sincerely yours,

HUANG, Yu-Tung

The point-to-point responses are as follows,

Summary and General Comments

Reviewer #1:

The current study was aimed to evaluate the association between Dengue virus (DENV) infection and major adverse cardiovascular events (MACEs, including acute myocardial infarction [AMI], heart failure and stroke). This is a population-based observation study. All laboratory confirmed dengue cases in Taiwan during 2009 and 2015 were included by Center for Disease Control (CDC) notifiable database. The primary outcome of the incidence of MACEs within one year of dengue was observed in 1,247 patients. They found that AMI, stroke and heart failure are significantly higher in the immediate time period (within one week) after dengue infection, especially in patients with >60 years of age, female gender and severe admission dengue cases. The authors concluded that cardiovascular events triggered by dengue virus are potentially preventable. Patients with dengue fever should be carefully monitored during the acute phase of disease to ensure early recognition of symptoms of AMI, stroke or heart failure. Whether dengue virus vaccination reduces the risk of MACEs in patients with a high risk of cardiovascular disease is an intriguing question.

This is an interesting study with some novel findings and clinical implications. The study was well-conducted. The conclusions were supported by the results. The manuscript was well-written. [Reply] Thank you.

Some points concerned:

1. On page 3, line 6: …. the "dengue associated of MACE" ("dengue-associated MACE" ?) should be taken into…
   [Reply] Revised, thanks. Please check the revised manuscript (Tracked version) page 3, line 49.
2. On page 3, line 10: … becomes a vivid "thread" ("threat"?) to non-tropical countries…
   [Reply] Revised, thanks. Please check the revised manuscript (Tracked version) page 3, line 54.
3. On page 4, line 7: …stroke, heart failure, arrhythmia and "cardiomyositis" ("myocarditis"?)
   [Reply] Revised, thanks. Please check the revised manuscript (Tracked version) page 4, line 67.
4. On page 9, line 6-7, (also in Tables 1, 3): ... we performed analyses in subgroups defined according to age group (0-40 years, "40-60 years" [41-60 years?], and >60 years), sex, …
   [Reply] We were sorry for this mistake. The right age group are 0-39, 40-59, and ≥60 years. We had corrected all this part, please check the revised manuscript (Tracked version) page 3, line 60; page 9, line 151; page 12, Table 1; page 14, line 194-195; and page 15, Table 3.

5. On page 14, line 10-11: …The risks of "ischemic stroke hemorrhagic stroke and heart failure" ("hemorrhagic stroke, ischemic stroke and heart failure"?) remained increased (IRR 4.33, 3.17, and 2.45, respectively), …
   [Reply] We have revised it, thanks. Please check the revised manuscript (Tracked version) page 14, line 201.

6. On page 19, line 4-5: …contractility and eventually leading ("to"?) decompensation…
   [Reply] Due to another reviewer's comment, we had rewritten this paragraph, thanks.

7. On page 19, line 10-11: …were frequent if "routine" electrocardiogram was "routinely" (redundant? Deleted?) performed for DENV-infected…
   [Reply] Thanks, this sentence has corrected as " A study in Brazil demonstrated that cardiovascular manifestations were common (19.7%), as arrhythmia (sinus bradycardia [13.8%], atrial [4.9%] and ventricular [4.0%] extrasystoles) was frequent if electrocardiogram was routinely performed for DENV-infected patients [25]." The word "routine" has deleted. Please check the revised manuscript (Tracked version) page 19, line 272-275.

8. On page 19, line 1 from the bottom: …if they attack in some specific patient groups, such as the "elder" ("elderly"?).
   [Reply] It should be "elderly", we have revised it, thanks. (page 20, line 281)

9. On page 20, line 2-3: …At higher "altitudes" (latitude?), cases of DENV infection are usually clustered during summer, …
   [Reply] Thank you, it should be "latitude". We have revised it. (page 20, line 284)

Reviewer #2:

The manuscript entitled "Major acute cardiovascular events after dengue infection– A population-based observational study" by Wei et al. is well written and reports interesting results. This study aimed to perform a large-scale evaluation of the association between Dengue virus infection and major adverse cardiovascular events. They observed significantly increased incidence rate ratio for major cardiovascular events (hemorrhagic stroke, ischemic stroke, acute myocardial infarction, and heart failure) mainly within the first week after the onset of Dengue virus infection. The study design is adequate and the results support its conclusions. However, some adjustments should be performed in the manuscript:

1. The text excerpt "DENV is taken up into macrophages…DENV-infected myotubes" (lines 252-259) was literally copied from the study cited by the reference 17, which is considered as plagiarism. Therefore, the aforementioned excerpt should be completely rewritten and all the manuscript should undergo a careful plagiarism check.

   [Reply] Thank you for the remind of this very important issue. Our original intention was to quote this entire passage. We totally agree with you that it may be considered as plagiarism. We have rewritten the paragraph and check the whole manuscript. Please check revised manuscript (Tracked version) page 18, line 252 to page 19, line 269. And we also checked our revised manuscript by Turnitin software. Please check our supplemental documents.

2. The reference 16 does not support the information from lines 220-222. Another study that supports the data from that excerpt should be chosen to replace the reference 16.

   [Reply] We thank the reviewer for pointing this out. The reference has been replaced with the proper citation. [reference 16] Margetic S. Inflammation and hemostasis. *Biochemia medica*. 2012; 22: 49-62.

3. I suggest the inclusion of a table describing the demographic characteristics of all the groups included in the study, including the individuals who had influenza and onychomycosis.

   [Reply] Thank you for this constructive suggestion. We add a Supplement Table describing the demographic characteristics of all the groups included in the study in the supplementary data.

   Due to the core aim of this study was focus on dengue infection and the study design was based-on self-control. Therefore, we did not provide the negative and positive controls' demographic characteristics. We agree that readers may also be interested in the demographic characteristics of control groups. However, in order to avoid improperly comparison for three groups, we chose to present their characteristics in the appendix Table.

Below are some language errors that should be eliminated:

1-Line 73: The word “Aedes” should be written in italics.

[Reply] Revised, thanks. Please check the revised manuscript (Tracked version) page 4, line 74.

2-Line 75: The word “attitudes” should be replaced with “latitudes”.

[Reply] Thank you, it should be "latitude". We have revised it. (page 4, line 76)

3-Line 110: The word “and” should be added before the word “NS1”.

[Reply] Revised, thanks. Please check the revised manuscript (Tracked version) page 7, line 109.

4-Line 200: A comma should be added between the words “ischemic stroke” and “hemorrhagic stroke”.

[Reply] Thank you. A comma has been added. Please check the revised manuscript (Tracked version) page 14, line 201.

Supplement Table. Baseline characteristics of patients with onychomycosis and influenza infection who had a MACE within the observation period

Characteristics	**Influenza**		**Onychomycosis**	
	N	%	N	%
Age				
0-39 years	196	4.29	245	1.66
40-59 years	627	13.72	2,733	18.48
≥60 years	3,743	81.90	11,812	79.86
Missing	4	0.09	0	0.00
Sex				
Male	2,609	57.09	9,115	61.63
Female	1,948	42.63	5,660	38.27
Hypertension	13	0.28	15	0.10
Diabetes mellitus	3,121	68.29	10,444	70.62
Dyslipidemia	1,857	40.63	5,652	38.22
MACE before observation period	1,050	22.98	4,347	29.39
Hemorrhage stroke	2,824	61.79	8,240	55.71
Ischemic stroke	165	3.61	623	4.21
Acute myocardial infarction	899	19.67	3,279	22.17
Heart failure	409	8.95	1,506	10.18
MACE after observation period	1,351	29.56	2,832	19.15
Hemorrhage stroke	3,328	72.82	10,097	68.27
Ischemic stroke	182	3.98	745	5.04
Acute myocardial infarction	769	16.83	3,464	23.42
Heart failure	519	11.36	2,061	13.94

MACE, major adverse cardiovascular events

20211108_MACEs after dengue infection [rev].docx

字數 2985

提交時間 11-11月-2021 10:58上午

文稿 ID 78694824

Major acute cardiovascular events after dengue infection– A population-based observational study

Abstract

Background

Dengue virus (DENV) infection may be associated with increased risks of major adverse cardiovascular effect (MACE), but a large-scale study evaluating the association between DENV infection and MACEs is still lacking.

Methods and findings

All laboratory confirmed dengue cases in Taiwan during 2009 and 2015 were included by CDC notifiable database. The self-controlled case-series design was used to evaluate the association between DENV infection and MACE (including acute myocardial infarction [AMI], heart failure and stroke). The "risk interval" was defined as the first 7 days after the diagnosis of DENV infection and the "control interval" as 1 year before and 1 year after the risk interval. The incidence rate ratio (IRR) and 95% confidence interval (CI) for MACE were estimated by conditional Poisson regression. Finally, the primary outcome of the incidence of MACEs within one year of dengue was observed in 1,247 patients. The IRR of MACEs was 17.9 (95% CI 15.80-20.37) during the first week after the onset of DENV infection observed from 1,244 eligible patients. IRR were significantly higher for hemorrhagic stroke (10.9, 95% CI 6.80-17.49),

ischemic stroke (15.56, 95% CI 12.44-19.47), AMI (13.53, 95% CI 10.13-18.06), and heart failure (27.24, 95% CI 22.67-32.73). No increased IRR was observed after day 14.

Conclusions

The risks for MACEs are significantly higher in the immediate time period after dengue infection. Since dengue infection is potentially preventable by early recognition and vaccination, the dengue-associated MACE should be taken into consideration when making public health management policies.

Author Summary

Dengue infection is the most rapidly spreading mosquito-borne viral illness worldwide and becomes a vivid threat to non-tropical countries. Previous research has documented that viral infection can increase risks of adverse cardiovascular events. There were sporadic reports about the association of cardiovascular events and dengue infection in the endemic tropical countries. Our study analyzed the risks for major adverse cardiovascular events, and found that acute myocardial infarction, stroke and heart failure were significantly higher in the immediate time period (within one week) after dengue infection, especially in patients with ≥60 years of age, female gender and severe admission dengue cases.

Introduction

Viral infection can directly and indirectly affect the cardiovascular system, resulting in increased risks of major cardiovascular events [1,2]. Many viruses have been reported with case reports of major adverse cardiovascular events (MACEs), including influenza, severe acute respiratory syndrome virus (SARS-CoV), cytomegalovirus, Epstein-Barr virus, influenza, SARS-CoV-2, etc [3-7]. Various MACEs have been recognized, including acute myocardial infarction, stroke, heart failure, arrhythmia and myocarditis. In a milestone study, respiratory viral infection, especially influenza, had a significant association with acute myocardial infarction [8]. Moreover, vaccinations are very important in public health. Studies on the effect of influenza and pneumococcal vaccines have shown a 17 to 36% reduction in cardiovascular events [9,10].

In the modern era, globalization and climate change bring new public health challenges, such as the COVID-19 pandemic and emerging tropical viral infections, such as dengue virus. Dengue virus (DENV) infection is the most rapidly spreading mosquito-borne viral illness worldwide, and it is transmitted by *Aedes* mosquitoes, mainly of the species *Aedes aegypti*. The epidemiology of DENV has changed due to global warming [11,12]. An increased number of DENV infections has been reported in places with higher altitudes.

Large-scale studies on the association of MACEs and DENV infection are still lacking. Although there were some studies reporting on the association of MACEs and DENV infection, most of these reports were on youth and children in tropical endemic regions. Because the MACEs and immune response following viral infection can be completely different in the youth and in adult populations, these findings may not be generalized for populations in the regions where most adults are DENV immunity-naïve.

To understand the association of MACEs and DENV infection, we conducted this study using two nationwide databases that provided data on unbiased laboratory-confirmed DENV infection cases. The results will provide evidences for helping manage high-risk patients and make public health policy for dengue infection. The association between DENV and MACEs may provide a scientific clue for managing the potential MACEs in other viral infections such as COVID-19 in the future.

Methods

Data Source

The data used in this study were retrieved from the National Health Insurance Research Database (NHIRD) and the Notifiable Disease Dataset of Confirmed Cases – Disease Prevention Database (NDDCC) under the Health and Welfare Data Center (HWDC) established by the

Ministry of Health and Welfare (MoHW) of Taiwan. Both the NHIRD and the NDDCC are official databases, and they are managed by the National Health Insurance Administration and Centers for Disease Control of Taiwan (TwCDC), respectively. All databases were encrypted due to privacy concerns but linkable for research purposes and limited to use at HWDC only. Detailed information and description of the study data and specification of HWDC were reported in a previous article [13].

Study Design and Populations

This is a population-based observation study. Data including all patients who had been initially diagnosed with dengue virus syndrome were collected from inpatient, emergency and outpatient department claims under the NHIRD between 2009 and 2015. We confirmed the diagnosis of DENV infection by obtaining their infection detection date (Id date) by linking to the NDDCC database. In Taiwan, all clinically suspected cases of DENV infection from physician offices, emergency departments, hospitals, long-term care facilities, and public health departments receive complete epidemiologic surveys and blood tests after being reported to the Taiwan Centers for Disease Control. The serology (IgM & IgG), real-time polymerase chain reaction (RT-PCR), and NS1 antigen were routinely examined for validation. Confirmed cases were all registered in the NDDCC database.

The hospitalization records for MACEs of laboratory-confirmed dengue cases were searched from the NHIRD inpatient dataset from 2008 to 2016. We included all subjects with MACEs one year before and after the DENV Id date. A schematic algorithm of the study is shown in Fig 1. If the MACEs were not related to DENV infection, then the incidence of MACEs for our selection subjects would presumably be distributed equally during the entire observation period. The first 7 and 14 days after the DENV Id date were defined as risk intervals, and the other periods were defined as control intervals. This study design was adapted from a previous study [8].

Due to potential concerns of bias, we added negative and positive control analyses. Onychomycosis, a nonviral and non-MACE-related infectious disease, was selected as our negative control. Infection with influenza virus served as our positive control. The analyses and research design for DENV infection and negative and positive controls were the same.

Definition

The DENV patients were identified by *International Classification of Diseases, Ninth Revision, Clinical Modification* (ICD-9-CM) codes 061 and 065.4 and *International Classification of Diseases, Tenth Revision* (ICD-10) code A90. MACEs, including hemorrhagic stroke (ICD-9-CM 430-432; ICD-10 I60-I62), ischemic stroke (ICD-9-CM 433-437; ICD-10 I63, I65-I67, G45, G46), acute myocardial infarction (AMI) (ICD-9-CM 410, 411; ICD-10 I20-I22,

I24), heart failure (ICD-9-CM 428; ICD-10 I50), and thromboembolic stroke (ICD-9-CM 415.1, 453.4; ICD-10 I26, I80.2), were selected. The comorbidities that were major risk factors for MACEs included hypertension (ICD-9-CM 401-405; ICD-10 I10-I16), diabetes mellitus (ICD-9-CM 250; ICD-10 E08-E13), and disorder of lipid metabolism (ICD-9-CM 415.1, 453.4; ICD-10 I26, I80.2) and were also included in our analysis.

Statistical Analyses

We calculated the incidence rate ratio (IRR) and 95% confidence interval (C.I.) for MACEs per 1,000 during the risk interval and the control interval via Poisson regression. The model accounted for multiple DENV exposures and hospitalization episodes for MACEs per DENV patient during the observation period. We also evaluated the risks during days 8-14, days 15-21, and days 22-28 post-DENV infection. In addition, we performed analyses in subgroups defined according to age group (0-39 years, 40-59 years, and ≥60 years), sex, severity level (admission or nonadmission case), and stratification by each individual MACE item.

Ethics Approval

This study was conducted in accordance with the Declaration of Helsinki and the Declaration of Taipei on ethical considerations regarding health databases by the World Medical Association. The study protocol was approved, and informed consent was exempted by the

Institutional Review Board of Chang Gung Medical Foundation (IRB No: 201901517B0) as all data converted from the original NHI's claim records were anonymized and due to tight regulations of on-site analysis at HWDC.

Results

There were 79,037 patient consultations for dengue virus infection in Taiwan from Jan 1, 2009, to Dec 31, 2015. Of these, 65,906 patients were laboratory-confirmed to have DENV infection. The primary outcome of the incidence of MACEs within one year of dengue was observed in 1,247 patients (Fig 2). Summary data for DENV-infected patients with cardiovascular and neurovascular events are shown in Table 1. A total of 856 (68.6%) DENV patients with MACEs were hospitalized. Most of the vascular events (78.35%) were observed in patients older than 60 years. A 53% (929 vs. 606) increase in the number of hospitalizations due to cardiovascular and neurovascular events within 1 year after the diagnosis of DENV infection was found. The major MACEs came from ischemic stroke and heart failure. Because three patients' ages were missing, we included only 1,244 patients divided into 2,488 episodes in the subsequent analysis.

Table 2 shows the IRR for MACEs after laboratory-confirmed dengue infection. These events were highest within 7 days after having dengue fever, and the IRR of MACEs was 17.9

(95% C.I. 15.80-20.37). The IRR was still higher in the second week after DENV infection (IRR=2.40, 95% C.I. 1.78-3.28), but there was no significant difference after the third (IRR=1.38, 95% C.I. 0.93-2.05) and fourth (IRR=1.10, 95% C.I. 0.71-1.71) week after DENV infection. A similar result was found in our positive control-influenza group. The first-week IRR for MACE was 15.76 (95% C.I. 14.88-16.70). On the contrary, the IRR was insignificant for onychomycosis (IRR=0.91, 95% C.I. 0.79-1.05).

In the subgroup analyses, the major results were consistent with the main analysis but with discrepant effects. These results are shown in Table 3. The MACEs were highest in patients ≥60 years of age (IRR=21.12, 95% C.I. 17.44-25.71) and lowest in patients 40-59 years old (IRR=6.61, 95% C.I. 4.29-10.16). Females had a higher risk of MACEs than males (IRR 21.17 for females and 15.94 for males). The MACE risk was nearly six times higher among admission cases than among non-admission cases (IRR 24.39 vs 4.17). All of the IRRs of MACEs from each disease were over 10 during the first week after DENV infection, and the IRRs were 10.9 for hemorrhagic stroke (95% C.I. 6.80-17.49), 13.53 for AMI (95% C.I. 10.13-18.06), 15.56 for ischemic stroke (95% C.I. 12.44-19.47), and 27.24 for heart failure (95% C.I. 22.67-32.73). The risks of ischemic stroke, hemorrhagic stroke and heart failure remained increased (IRR 4.33, 3.17, and 2.45, respectively), but insignificance was observed among AMI patients in the second week of DENV infection.

Discussion

Our study found that the risk of MACEs was increased in patients within the early two weeks of DENV infection. The possible mechanisms for increased MACEs following DENV infection are unclear and complex. Many proposed viral, host response and immune mechanisms are known to be involved in the cardiac and vascular manifestations of DENV infections.

Viral infection can directly and indirectly augment cardiovascular risks [1,2]. DENV can cause direct infection of the cardiovascular system. DENV-induced myocarditis and myopathy have been reported, as DENV antigen was seen in cardiac tissues, including cardiomyocytes, myocardial interstitial cells, and endothelial cells [14]. DENV-infected vascular endothelium may also be responsible for both endothelial leakage and a longer effect on endothelial function. Children and adolescents with a history of dengue hemorrhagic fever have been reported to have an increased carotid intima-media thickness [15].

The host inflammatory response to infection often results in the release of proinflammatory cytokines and the activation of platelets, leukocytes, and endothelial cells that can activate procoagulant pathways while inhibiting anticoagulant pathways [16]. Vasoactive and proinflammatory cytokines increase capillary leakage, and endothelial dysfunction may also cause myocardial dysfunction. Altered intracellular calcium homeostasis potentially causes

electrical abnormalities that cause arrythmia or desynchronized myocardial movement and eventually cause cardiovascular events [17].

Vascular thrombosis is a major complication of infection and may be related to the pathogenesis of DENV-related MACEs [3,4]. Case reports of virus-associated thrombosis have been reported in many kinds of viruses, such as SARS-CoV-2, cytomegalovirus, Epstein-Barr virus, influenza, etc [3-7]. DENV infection is known for its hemorrhagic features but has also been reported in some cases of major thromboembolic events, including ischemic stroke, deep vein thrombosis, pulmonary embolism, central vein thrombosis, and mesenteric vein thrombosis [7,18-20].

Platelet dysfunction may play a crucial role in DENV-associated ischemic stroke and myocardial infarctions. Although thrombocytopenia is a common presentation of DENV infection, rebound thrombocytosis has also been reported [21]. Thrombocytosis may increase the risk of thromboembolic events, such as myocardial infarction, stroke, venothromboemboli and congestive heart failure.

Risks for cardiovascular events such as myocardial infarction or stroke have been reported to increase within 180 days after bacteremia and influenza infection [22]. In our study, DENV-associated thrombotic events mostly occurred within the first 2 weeks of illness and were highest

within the first 7 days. Previous reports on DENV-associated thrombosis showed similar findings [19,23]. Some patients with no risk factors for thrombosis, such as smoking, obesity or use of contraceptives, have thrombotic events during the first 5 days of DENV [19]. Most reported cases of DENV-associated thrombosis were seen in patients 31-60 years of age [7,19,23,24]. Our patients were mostly male and more than 40 years of age and had a medical history of hypertension, diabetes mellitus and hyperlipidemia (67.28%, 38.49%, and 34.96%, respectively). Li et al reported that the incidence of stroke in DENV infection was higher in male patients with comorbidities. They concluded that these factors are strong risk factors that may mask the effect of dengue fever on the occurrence of stroke [20].

In addition to thromboembolic events, heart failure was significantly increased. DENV can affect heart function via different mechanisms. The virus is taken in by macrophages, causing the activation of T cells and subsequent release vasoactive and pro-inflammatory cytokines. The release of these cytokines leads to capillary leakage and possible myocardial damage [17]. The interaction between NS1 protein and vascular endothelial glycocalyx layer is thought to increase capillary permeability. The resulting plasma leakage can result to myocardial interstitial edema and cause cardiac dysfunction by reducing preload and alteration of coronary microcirculation. Changes in intracellular calcium homeostasis in DENV-infected myotubes could lead to contractile dysfunction and ultimately to decompensation of heart failure [17].

DENV-associated MACEs may be underreported because many patients are asymptomatic or mildly symptomatic. Taking abnormal cardiovascular rhythm as an example, mild arrhythmia is common and frequently ignored. A study in Brazil demonstrated that cardiovascular manifestations were common (19.7%), as arrhythmias (sinus bradycardia [13.8%], atrial [4.9%] and ventricular [4.0%] extrasystoles) were frequently observed if electrocardiogram was routinely performed for DENV-infected patients [25]. Five percent of DENV patients had prolongation of the QTc interval [26]. In a DENV outbreak of 2005 in Sri Lanka, ECG abnormalities were found in 62.5% of patients (the median age: 34, ranging 12 to 76 years old), and 80% of them developed transient hypotension and tachycardia-bradycardia syndrome suggestive of transient severe cardiac dysfunction [27]. These may be associated with significant morbidity and higher mortality if they affect specific high-risk patient groups, such as the elderly or patients with underlying cardiac conditions.

Limitations

There are some limitations in this study. At higher latitudes, cases of DENV infection are usually clustered during summer, which may cause bias because the distribution of MACE incidence is affected by the season. However, there was a small seasonal variation in endemic

DENV in Taiwan. Moreover, based on our self-controlled design including one year before and after, the effect of seasonal variation should be eliminated.

Laboratory data, such as platelet count and coagulation status, were lacking in the database. Information about smoking, diet, body mass index and daily activity was not recorded in the NHIRD. Ideally, similar viral infections should be chosen as controls. Patients infected with influenza served as positive controls as influenza has been known to increase the risk of MACEs. However, it was not possible to choose a viral disease that is a CDC-reportable infectious disease and has been proven not to cause MACEs. Thus, onychomycosis, a common localized dermatophyte infection without known systemic infection, was chosen as the negative control. In this study, we did not analyze the different serotypes of DENV, as in the past endemic of 2014-2015 almost all cases were DENV-1 and DENV-2 infections.

Finally, the majority of exposure dates were based on the date of disease onset. It is possible that the date of disease onset was inaccurate in some cases as a result of patient recall bias and error in data entry. However, the Communicable Disease Control Act of Taiwan demands physicians to formally report a case of dengue infection to the government authority within 24 hours if the diagnosis of dengue fever is suspected. Although there is probably an error of a few

days in some cases, the risk period was measured in weeks in this study, hence the discrepancy is not expected to significantly affect the results.

Conclusion

In conclusion, our study found that MACEs are associated with acute DENV infection, which is important because cardiovascular events triggered by dengue virus are potentially preventable by early recognition, public health management and vaccination. The risk for MACEs, including AMI, heart failure and stroke, is significantly higher in the immediate time period after dengue virus infection. Patients with dengue fever should be carefully monitored during the acute phase of disease to ensure early recognition of symptoms of impending AMI, stroke or heart failure. Influenza vaccines have been proven to reduce cardiovascular events, and the benefit is even greater in the population with known coronary artery disease [9,10]. Whether dengue virus vaccination reduces the risk of MACEs in patients with a high risk of cardiovascular disease is an intriguing question.

20211108_MACEs after dengue infection [rev].docx

原創性報告

16%
相似度指數

主要來源

1	www.ncbi.nlm.nih.gov<br>網際網路	134 字 — 4%
2	www.nejm.org<br>網際網路	74 字 — 2%
3	Anne-Marie Connolly-Andersen, Edvin Hammargren, Heather Whitaker, Mats Eliasson, Lars Holmgren, Jonas Klingström, Clas Ahlm. "Increased Risk of Acute Myocardial Infarction and Stroke During Hemorrhagic Fever With Renal Syndrome", Circulation, 2014<br>Crossref	52 字 — 1%
4	Yacoub, Sophie, Heiman Wertheim, Cameron P. Simmons, Gavin Screaton, and Bridget Wills. "Cardiovascular manifestations of the emerging dengue pandemic", Nature Reviews Cardiology, 2014.<br>Crossref	28 字 — 1%
5	link.springer.com<br>網際網路	28 字 — 1%
6	www.mysciencework.com<br>網際網路	27 字 — 1%
7	www.astmh.org<br>網際網路	23 字 — 1%

8	Hao-Ming Li, Ying-Kai Huang, Yuan-Chih Su, Chia-Hung Kao. "Risk of stroke in patients with dengue fever: a population-based cohort study", Canadian Medical Association Journal, 2018<br>Crossref	22 字 — 1%
9	You_Ming Chen, Yuan Kao, Chien_Chin Hsu, Chia_Jung Chen et al. "Real_time interactive artificial intelligence of things–based prediction for adverse outcomes in adult patients with pneumonia in the emergency department", Academic Emergency Medicine, 2021<br>Crossref	18 字 — 1%
10	stage.uspharmacist.com<br>網際網路	17 字 — < 1%
11	www.science.gov<br>網際網路	17 字 — < 1%
12	www.karger.com<br>網際網路	14 字 — < 1%
13	Ganesh Asaithambi, Emily H. Marino, Bridget M. Ho, Megan E. Tipps. "Outcomes of Homeless Ischemic Stroke Patients Receiving Intravenous Thrombolysis in the United States", Journal of Stroke and Cerebrovascular Diseases, 2021<br>Crossref	13 字 — < 1%
14	www.elsevier.es<br>網際網路	13 字 — < 1%
15	en.wikipedia.org<br>網際網路	12 字 — < 1%

16	Emelia J. Benjamin, Michael J. Blaha, Stephanie E. Chiuve, Mary Cushman et al. "Heart Disease and Stroke Statistics—2017 Update: A Report From the American Heart Association", Circulation, 2017<br>Crossref	9 字 — < 1%
17	bmcgastroenterol.biomedcentral.com<br>網際網路	9 字 — < 1%
18	mdedge-files-live.s3.us-east-2.amazonaws.com<br>網際網路	9 字 — < 1%
19	weekly.chinacdc.cn<br>網際網路	9 字 — < 1%
20	M.-C. KO, C.-C. LIU, C.-K. LIU, L.-C. WOUNG, H.-F. CHEN, H.-F. SU, C.-Y. LI. "Incidence of renal and perinephric abscess in diabetic patients: a population-based national study", Epidemiology and Infection, 2010<br>Crossref	8 字 — < 1%
21	jaha.ahajournals.org<br>網際網路	8 字 — < 1%
22	www.hindawi.com<br>網際網路	8 字 — < 1%
23	www.mdpi.com<br>網際網路	8 字 — < 1%
24	Hook, Sarah A.. "Public Health Considerations for a Potential Lyme Disease Vaccine in the United States: Cost of Illness, Vaccine Acceptability, and Net Costs of a Vaccination Program", Colorado State University, 2021<br>ProQuest	7 字 — < 1%
25	digitalcommons.odu.edu	

網際網路

7 字 — < 1%

不含引用資料	關閉	排除相符處	關閉
不含參考書目	關閉		
